# The Antibacterial Activity of *Rhazya stricta* Extracts against *Klebsiella pneumoniae* Isolated from Some Soil Invertebrates at High Altitudes

**DOI:** 10.3390/molecules28083613

**Published:** 2023-04-21

**Authors:** Mohamed M. Hassan, Bander Albogami, Tarombera Mwabvu, Mohamed F. Awad, Roqayah H. Kadi, Alaa A. Mohamed, Jamal A. Al-Orabi, Montaser M. Hassan, Mohsen Mohamed Elsharkawy

**Affiliations:** 1 Department of Biology, College of Science, Taif University, P.O. Box 11099, Taif 21944, Saudi Arabia; 2 High Altitude Research Centre, Taif University, P.O. Box 11099, Taif 21944, Saudi Arabia; 3 School of Biology & Environmental Sciences, University of Mpumalanga, Private Bag X 11283, Mbombela 1200, South Africa; 4 Department of Biology, Faculty of Science, University of Jeddah, Jeddah 21959, Saudi Arabia; 5 Department of Agricultural Botany, Faculty of Agriculture, Kafrelsheikh University, Kafr Elsheikh 33516, Egypt

**Keywords:** biodiversity, biofilm, *Rhazya stricta*, *Klebsiella pneumoniae*, soil invertebrates, Saudi Arabia

## Abstract

*Klebsiella* is a common dangerous pathogen for humans and animals and is widely present in the digestive system. The genus *Klebsiella* is ubiquitous, as it is endemic to surface water, soil, and sewage. In this study, 70 samples were obtained from soil-dwelling invertebrates from September 2021 to March 2022 from Taif and Shafa in different altitudinal regions of Saudi Arabia. Fifteen of these samples were identified as *Klebsiella* spp. The *Klebsiella* isolates were genetically identified as *Klebsiella pneumoniae* using rDNA sequencing. The antimicrobial susceptibility of the *Klebsiella* isolates was determined. Amplification of virulence genes was performed using PCR. In this study, 16S rDNA sequencing showed a similarity from 98% to 100% with related *K. pneumonia* from the NCBI database, and the sequences were deposited in the NCBI GenBank under accession numbers ON077036 to ON077050. The growth inhibition properties of ethanolic and methanolic extracts of the medicinal plant *Rhazya stricta*’s leaves against *K. pneumoniae* strains using the minimum inhibitory concentration (MIC) method and disc diffusion were evaluated. In addition, the biofilm inhibitory potential of these extracts was investigated using crystal violet. HPLC analysis identified 19 components divided into 6 flavonoids, 11 phenolic acids, stilbene (resveratrol), and quinol, and revealed variations in the number of components and their quantities between extracts. Both extracts demonstrated interesting antibacterial properties against *K. pneumoniae* isolates. The 2 extracts also showed strong biofilm inhibitory activities, with percentages of inhibition extending from 81.5% to 98.7% and from 35.1% to 85.8% for the ethanolic and methanolic extracts, respectively. *Rhazya stricta* leaf extract revealed powerful antibacterial and antibiofilm activities against *K. pneumoniae* isolates and could be a good candidate for the treatment or prevention of *K. pneumonia*-related infections.

## 1. Introduction

The gastrointestinal tract of invertebrates is an ideal location for microflora; the stomach is rich in bacteria, while the proctodaeal region is rich in fungi [1]. From the hindgut of *Glomeris* species, six bacteria, six actinomycetes, and two fungal strains were isolated [2]. *Pseudomonas stutzeri* and *Pseudomonas putida* survive passage through the gut of a millipede, *Pachyiulus flavipes*, and increase fresh excrement [3]. Bacteria recovered from the gut of millipedes (*Ommatoiulus sabulosus*) include *Klebsiella*, *Bacillus*, and *Corynebacterium* species, while actinomycetes (such as *Micromonospora* sp.) are known to accumulate in the hindgut and are likely to participate in the breakdown of chitin [4]. Fecal pellets consist of dense populations of micro-organisms [2]. The genus *Klebsiella*, a severe opportunistic pathogen belonging to the family Enterobacteriaceae, is a major pathogen associated with urinary, respiratory, gastrointestinal, and skin infections in humans [5]. *Klebsiella pneumoniae* control is impaired by the frequent multidrug-resistant phenotype and genotype, representing a major threat to neonates, the elderly, and immuno-compromised patients [6,7]. *Klebsiella* is ubiquitous in terms of habitat association. It is also a resident or transient flora, particularly in the gastrointestinal tract of some invertebrates [2]. In addition, *Klebsiella* species frequently acquire antibiotic resistance genes for all classes of antibiotics and are considered the first microorganism to help in spreading resistance and virulence genes [8,9,10]. Similar to other opportunistic pathogens, *K. pneumoniae* is a ubiquitous bacterium that thrives in environmental compartments (e.g., soil, plants, and waterways) [11,12]. While sometimes *K. pneumoniae* bacteria are present in human and animal waste sources and, therefore, can be considered environmental pollutants, at other times, these *K. pneumoniae* strains are environmental strains that appear in their natural habitat [12,13]. Water, vegetation, and soil have been described as the native environments for *K. pneumoniae* [2]. Phenotypic and genotypic traits were compared between isolates of *K. pneumoniae* obtained from hospitals and those obtained from the natural environment [14]. *K. pneumoniae* is a prominent hospital-acquired pathogen, as well as a significant foodborne pathogen that may cause liver abscesses, pneumonia, septicemia, and diarrhea [15,16,17]. *K. pneumoniae* has been recognized as a major food-borne pathogen due to its prevalence outside of the medical environment, where it is often detected in cooked meals, raw vegetables, powdered infant formula, fish, meat, and street foods [18,19,20,21,22,23]. It is important to investigate the common and distinct genomic traits of clinical and environmental strains of *K. pneumoniae* [24]. Although the distinction between clinical strains and environmental strains is difficult, the characterization of strains from both origins is crucial to assess the harmful effect of both types and to study the evolution and acquisition of new genetic traits from one source to the other, as well as to infer pathways of transmission from the environment to humans [14].

*K. pneumoniae* isolates were subjected to a medicinal plant extract, *Rhazya stricta*, which is an economically important medicinal plant. In Saudi Arabia and many Asian countries, *R. stricta* and its metabolites are traditionally used for the treatment of cancer, skin diseases, hypertension, rheumatism, sore throat, syphilis, parasitic infections, inflammatory conditions, and fever [25,26]. Various parts of *R. stricta* contain many phytochemical constituents, such as alkaloids, flavonoids, triterpenes, and volatile bases [25,27], which display potential antimicrobial and biological activities [25]. Furthermore, leaf and fruit extracts of *R. stricta* have shown antimicrobial properties against many multidrug-resistant human pathogens [26,28]. However, the antibiofilm activity of *R. stricta* has not been explored.

In the present study, *K. pneumoniae* strains were isolated from invertebrate animals collected from different regions of Taif in Saudi Arabia. Therefore, the main aim of this study was to classify and characterize *K. pneumoniae* isolates obtained from invertebrate animals, and to evaluate the antibacterial and antibiofilm properties of the phenolic components in *R. stricta* leaf ethanolic and methanolic extracts against *K. pneumoniae* isolates and their virulence gene profiles.

## 2. Results

### 2.1. Isolation and Identification of K. pneumoniae Isolates

#### 2.1.1. Isolation of *K. pneumoniae* Isolates

Fifteen isolates were obtained from different invertebrate animals (millipedes and isopods) collected from the Taif governorate and identified as *K. pneumoniae*. The location and invertebrates are presented in Table 1. Nine *K. pneumoniae* isolates were obtained from millipede guts, three of which (KTU-10, KTU-11, and KTU-12) were collected from Wady Ghazal, Taif, and six (KTU-1, KTU-2, KTU-3, KTU-13, KTU-14, and KTU-15) from Al-Shafa, Taif. Six *K. pneumoniae* isolates (KTU-4, KTU-5, KTU-6, KTU-7, KTU-8, and KTU-9) were isolated from the gut of an isopod, *Porcellio laevis*, collected from the Taif University Garden in Hawia, Taif, Saudi Arabia.

#### 2.1.2. Molecular Genotyping of *K. pneumoniae*

**Figure 1:**
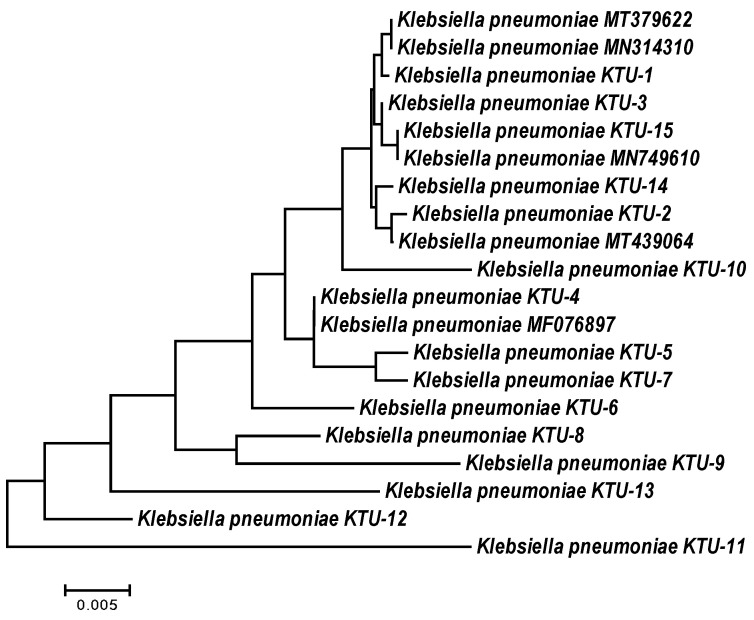
Neighbor-joining phylogeny tree based on 16S rDNA gene sequences of *Klebsiella pneumoniae* isolates collected from some invertebrates in Taif, Saudi Arabia, with 1000 bootstraps.

The 16S rRNA gene of all *K. pneumoniae* isolates was amplified and sequenced, and specific fragments were aligned and compared with the available 16S rRNA sequences for other *K. pneumoniae* isolates in the NCBI database. The sequences of the *K. pneumoniae* isolates were deposited in the NCBI GenBank under accession numbers ON077036 to ON077050. The BLAST results showed that the partial 16S rRNA sequences were more similar to other sequences from the NCBI database. The similarity matrix among the *K. pneumoniae* isolates and related strains from the NCBI database ranged from 98 to 100%. For example, the *K. pneumoniae* KTU-11 isolate with accession number ON077046 has low similarity to *K. pneumoniae* strains. The *K. pneumoniae* KTU-1 isolate with accession number ON077036 is moderately similar to the *K. pneumoniae* strain MT-379622 and the *K. pneumoniae* strain MN-314311. The *K. pneumoniae* KTU-15 isolate with accession number ON077050 has high similarity to the *K. pneumoniae* strain MN749610, with approximately 100% similarity (Table 2, Figure 1). 

#### 2.1.3. Antimicrobial Susceptibility

*Klebsiella pneumoniae* was tested for antimicrobial susceptibility to 12 types of antibiotics. The overall susceptibility, intermediate susceptibility, and resistance values were determined (Table 3). Most *K. pneumoniae* strains showed a high percentage of resistance to carbecillin (100%), oxacillin (100%), cefoxitin (100%), amoxicillin (100%), and penicillin (93.3%). Erythromycin (80%), amkacillin (53.3%), ampicillin (40%), and cefrizine (40%) indicated moderate susceptibility. On the other hand, intermediate resistance was found to sulfamethoxazole/Trimethoprim (26.7%). Moreover, all the *K. pneumoniae* isolates were sensitive to ciprofloxacin and gentamicin. 

#### 2.1.4. Detection of Virulence Genes in *K. pneumoniae*

**Figure 2:**
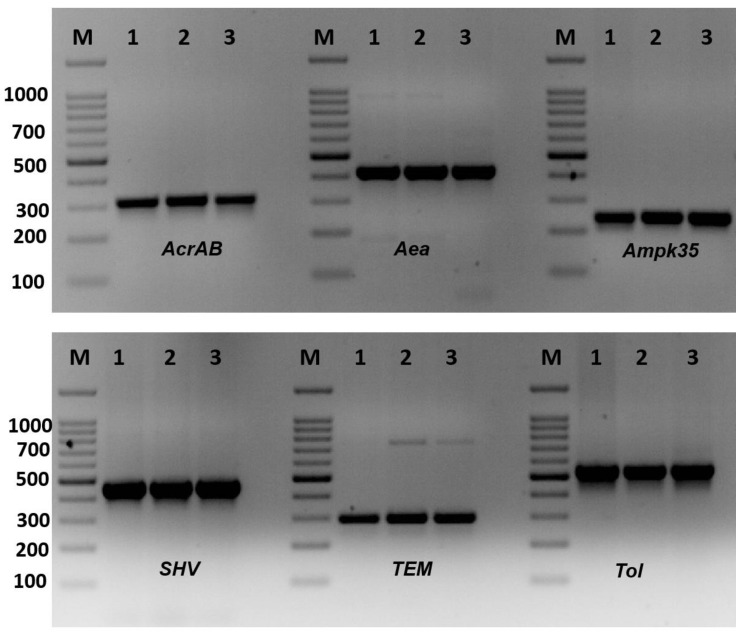
Amplification of virulence genes of *Klebsiella pneumoniae* isolates by single PCR. Amplification of *AcrAB* gene (312 bp), amplification of *Aea* gene (410 bp), amplification of *Ompk35* gene (241 bp), amplification of *SHV* gene (436 bp), amplification of *TEM* gene (295), and amplification of *Tol* genes (527). M: 100 bp DNA ladder, 1–3: some positive isolates.

The existence of antibiotic-resistant genes is shown in Figure 2 and Table 4. The virulence genes *AcrAB, mdtk, OmpK35, FimH*, and *RmpA* were recorded in all *K. pneumoniae* isolates (Table 4). The *K1* gene, which is responsible for the formation of capsule and K genotypes, was found in only 3 isolates of *K. pneumoniae*, KTU-7, KTU-8, and KTU-11, representing 15% of the isolates. The *K. pneumoniae* KTU-8 and KTU-10 isolates have the most investigated virulence genes. *OmpK35* plays a role in K. pneumoniae infection and virulence. The Aea gene was found in all *K. pneumoniae* isolates, except KTU-5, KTU-8, KTU-9, and KTU-11. *TolC* was also found in all *K. pneumoniae* isolates, except *K. pneumoniae* KTU-9 and KTU-11. Moreover, the *SHV* and *TEM* genes were found in all *K. pneumoniae* isolates, whereas the *CTX* gene was found in two isolates, *K. pneumoniae* KTU-8 and KTU-10.

### 2.2. The Potential of R. stricta Extract against K. pneumoniae

#### 2.2.1. Chemical Composition of *R. stricta* Leaf Extracts

The chemical composition of the ethanolic and methanolic extracts of *R. stricta* are listed in Table 5. Nineteen components were obtained from the HPLC analysis of these extracts; they were divided into six flavonoids, eleven phenolic acids, stilbene (resveratrol), and quinol. In total, 17 compounds were detected in each extract, with a quantity of 15,292.89 mg/kg and 33,050.65 mg/kg for the ethanolic and methanolic extracts, respectively, indicating that the methanolic extract is richer in phenolic compounds than the ethanolic extract. 

The results revealed variations between the two extracts in terms of the number and quantity of components. The major compounds of *R. stricta* ethanolic extract are Quinol, Resveratrol, p-Coumaric acid, Benzoic acid, Rutin, Quercitin, Myricetin, and Kaempferol. However, the major compounds of methanolic extract are Resveratrol, Benzoic acid, Ferulic acid, Rutin, Quercitin, Neringein, and Kaempferol. Both *R. stricta* extracts are rich in flavonoids (11,104.27 mg/kg and 21,357.93 mg/kg for the ethanolic and methanolic extracts, respectively) compared to other phenolic compounds (4188.62 mg/kg and 11,692.72 mg/kg for the ethanolic and methanolic extracts, respectively).

#### 2.2.2. Antibacterial Activity of *R. stricta* Extracts against *K. pneumoniae*

##### Disc Diffusion

The ethanolic and methanolic extracts of *R. stricta* leaves were examined for their antimicrobial activity against *K. pneumoniae* isolates (Table 6). First, the disc diffusion method showed that the two extracts were active against all isolates, despite the variation in the type of inhibitory action. *R. stricta* ethanolic extract demonstrated strong inhibitory activity against 40% of the strains, compared to the methanolic extract, which showed a strong inhibitory action on 33.3% of the isolates. As shown in Table 6, the ethanolic extract was slightly more effective than the methanolic extract against *K. pneumoniae* isolates.

##### Determination of (MIC) and (MBC)

The antimicrobial activities of the ethanolic and methanolic extracts of *R. stricta* leaves were also investigated using MIC and MBC for the 15 *K. pneumoniae* isolates. For the ethanolic extract, the MIC ranged from 0.122 to 0.970 mg/mL, whereas the MBC ranged from 0.224 mg/mL to 1.9 mg/mL. For the methanolic extract of *R. stricta* leaves, the MIC values varied from 0.224 mg/mL to 1.9 mg/mL, while the MBC values ranged from 0.448 mg/mL to 3.9 mg/mL. Accordingly, the ethanolic extract had the greatest antibacterial activity against *K. pneumoniae* isolates compared with the methanolic extract. 

### 2.3. Biofilm Formation and Inhibition

#### 2.3.1. Biofilm Formation on Polystyrene Surface

The bacterial isolates were inspected for their ability to produce biofilms on polystyrene surfaces (Table 7). The results showed that all *K. pneumoniae* strains were capable of producing biofilms and were allocated as follows: 26.7% were highly positive biofilm producers, with OD570 values varying from 1.015 to 1.060, and 73.3% were low-grade positive, with OD570 values ranging from 0.442 to 0.808.

#### 2.3.2. Biofilm Inhibition

The ability of *R. stricta* ethanolic and methanolic extracts to inhibit biofilm formation by *K. pneumoniae* isolates is shown in Table 7. Isolates showing potential for biofilm formation were selected for this experiment. Fifteen strains were classified as low-grade and highly positive biofilms, and both extracts demonstrated strong biofilm inhibition activity. 

#### 2.3.3. Antibiofilm Activity

The present investigation revealed that the ethanolic extract of *R. stricta* leaves has strong biofilm inhibition activity on all the isolates (15 strains), with the percentage of inhibition extending from 81.5% to 98.7%. Overall, 4 out of 5 highly positive isolates (80%) were biofilm-negative. In addition, 10 low-grade positive isolates (75%) changed to biofilm-negative after treatment. 

Biofilm inhibitory activities were also observed for the methanolic extract, with most isolates ranging from 35.1% to 85.8%. Furthermore, the same results observed for the four highly positive biofilm isolates treated with the ethanolic extract were obtained after treatment with the methanolic extract. However, 4 low-grade positive isolates (26.7%) were biofilm-negative. Table 7 shows that isolate No. 10 conserved its initial biofilm phenotype after treatment with the 2 extracts, despite the large decrease in the amount of biofilm; however, the methanolic extract did not affect the ability of isolate No. 1 to form a biofilm. No significant correlation was detected between the MIC and antibiofilm activity for either the methanolic or the ethanolic extract of *R. stricta* leaves. 

## 3. Discussion

Recently, 16S rRNA gene sequencing has been used as an alternative method for the molecular detection of various microbes, including *K. pneumoniae* [4]. This gene is found in all bacteria and, hence, ensures the accurate identification of bacteria at the genus and species levels [29]. Thus, sequencing can be reasonably applied to the preparation of many microbes, especially those isolated from the external environment or from other animals. In the present study, 16S rRNA gene sequencing displayed similarities between *K. pneumoniae* isolated from invertebrates and those obtained from GenBank, indicating that sequencing has the potential to be more sensitive than culture-dependent morphological and microscopic identification [30]. 

*Klebsiella pneumoniae* is a public health problem worldwide. This bacterium is the most prominent antibiotic-resistant acquired pathogen. Infections can spread from person to person through the respiratory system, the environment, or by using contaminated medical equipment [4]. Therefore, the discovery of new therapeutic agents, especially natural products, against *K. pneumoniae* is highly important.

Currently, plant compounds have emerged as potential candidates, given the interest of scientists to search for antimicrobial and antibiofilm drugs. Among these, *R. stricta* has gained attention because of its medicinal uses [25]. In this study, the potential antibacterial properties of ethanolic and methanolic extracts of *R. stricta* against *K. pneumoniae* isolated from invertebrates were investigated. The isolates were investigated by growth inhibition assays. Experiments showed that the extracts of *R. stricta* leaves have strong antibacterial activity [27,31]. 

The high ability of *R. stricta* leaf extracts to prevent the growth and multiplication of this bacterium, observed in this study, may be attributed to the phenols and flavonoid compounds found in these extracts [32,33]. It has been shown that flavonoids, such as quercetin [32], kaempferol, and catechin [26], exhibit great growth inhibition activity against *K. pneumoniae* isolates. Flavonoids, which are the major components of these extracts, are responsible for the inhibition of nucleic acid synthesis [26], damage to the cytoplasmic membrane through the alteration of its function [32,33], inhibition of energy metabolism by the alteration of the cytoplasmic membrane, and inhibition of the energy supply for bacteria [26]. In addition, the inhibition of cell membrane synthesis and the aggregatory effect on whole bacterial cells have also been reported [31]. Several studies have demonstrated the antibacterial properties of phenolic acids, especially caffeic acid, ferulic acid, coumaric acid, and chlorogenic acid, which have antibacterial activities [34,35]. Phenolic acids damage the *K. pneumoniae* cell wall, leading to cytoplasmic leakage and changes in bacterial cell morphology [26,34,35]. Moreover, the high *K. pneumoniae* growth inhibition activity seems to be due to the synergetic effect of flavonoids and other phenolic compounds present in the *R. stricta* leaf extracts. 

In the present study, the ethanolic extract of *R. stricta* leaves was more effective against *K. pneumoniae* isolates than the methanolic extract, despite its lower abundance of flavonoids and phenols. This can be attributed to quinol and chlorogenic acid, which do not exist in the methanolic extract, and/or to myricetin and p-Coumaric acid, which are more abundant in the ethanolic extract. Accordingly, Xie et al. [36] reported that myricetin displayed the most significant antimicrobial activity of all the flavonoids and exhibited extensive activity against *K. pneumoniae* and many other pathogenic bacteria [26,37]. Furthermore, myricetin inhibits *Escherichia coli* DnaB helicase, which plays a major role in DNA replication and elongation [38]. In addition, p-Coumaric acid effectively inhibited the growth of *K. pneumoniae* and other pathogenic bacteria. p-Coumaric acid is responsible for the disruption of bacterial cell membranes and the inhibition of cellular functions by binding to bacterial genomic DNA [38]. Ma et al. [39] reported that quinol exhibited relatively strong antibacterial activity against *K. pneumoniae* by destroying the bacterial cell membrane and cell wall, increasing permeability, and influencing the expression of genes. However, chlorogenic acid does not show significant antibacterial activity [26]. 

*Klebsiella pneumoniae* isolates were examined for their ability to develop biofilms on polystyrene surfaces, and the experiment demonstrated that 23.33% of the isolates were strong biofilm producers, while 50% were low-grade positive producers. This finding demonstrates the high potential of *K. pneumoniae* strains to produce biofilms, confirming that *K. pneumoniae* is the most prevalent bacterium in biofilm-associated infections [40]. Biofilm, as an important virulence factor, is responsible for more than 65% of nosocomial infections and 80% of microbial infections [41]. Biofilms are associated with nasal colonization of the respiratory system, endocarditis, soft tissue infections, urinary tract infections, and other diseases [4]. Biofilms are also a severe issue in the field of urology because they are responsible for the persistence of bacteria in the genitourinary tract over the long term [37]. *K. pneumoniae* biofilms have been associated with medical equipment and chronic infections, and the presence of biofilms makes bacteria more resistant to antibiotics and phagocytosis, making their treatment more difficult [37]. Therefore, the discovery of novel therapeutic strategies for biofilm inhibition is important. Extracts of *R. stricta* leaves were tested for their ability to inhibit biofilm formation by *K. pneumoniae*. Antibiofilm examination showed that both plant extracts displayed strong biofilm inhibitory activity, with a 98.7% reduction in the amount of biofilm produced. This activity is largely due to flavonoids as a major component, in addition to other phenolic compounds found in the extracts. This result emphasizes the findings of Nielsen et al. [37], who reported that flavonoids are responsible for the reduction of bacterial adhesion, biofilm formation, and the inhibition of quorum sensing (cell-to-cell communication system in the biofilm formation signal receptors TraR and RhlR). Furthermore, a decrease in the amount of biofilm could be considered a reduction in *K. pneumoniae* virulence, which is in agreement with Saadatian et al. [42], who mentioned that flavonoids inhibit bacterial virulence factors. Moreover, Xie et al. [33] showed that flavonoids, such as quercetin, kaempferol, naringenin, and apigenin, suppress the activity of autoinducer-2, which is responsible for cell-to-cell communication and, consequently, reduces biofilm synthesis. In this study, the ethanolic extract also displayed biofilm inhibitory properties more than the methanolic extract, in addition to its growth inhibition activity, indicating that the components involved in growth inhibition are the same as those associated with biofilm inhibition, and that myricetin inhibits biofilm formation by *K. pneumoniae* [36]. Additionally, Saadatian et al. [42] revealed that flavonoids efficiently inhibited the bacterial biofilm matrix by targeting Bap-like amyloids. Myricetin also inhibits curli-dependent biofilm formation in *E. coli* [37]. 

Deletion of *OmpK36* or *OmpK35*/*OmpK36* can reduce the virulence of highly contagious *K. pneumoniae* strains and increase their susceptibility to neutrophil phagocytosis [43]. In our study, *OmpK35* porin-coding genes were simultaneously detected in all *K. pneumoniae* isolates. A direct correlation between efflux pumps and the virulence of pathogenic bacteria was reported by Padilla et al. [44]. Most strains of intestinal bacteria contain genes that encode iron absorption systems, such as enterochelin or aerobactin. Iron plays an important role, as it can inhibit T-cell proliferation, in addition to promoting iron absorption. The *rmpA*,* wabG*,* uge*,* Ycfm*,* fimh1*,* EntB*,* Ybt-irp2*, and *kfu* genes have been reported in most antibiotic-resistant *K. pneumoniae* isolates [43]. The most pathogenic genes lead to high-pathogenicity strains that contain virulence genes prevalent in *Klebsiella* species [5]. ESBLs are now found in all Enterobacteriaceae species worldwide [45]. The ESBL genes *TEM* and *SHV* were found in all *K. pneumoniae* isolates in this investigation, and only three of them harbored the CTX gene. The number of CTX-M-producing *K. pneumoniae* strains has also increased [5,45].

## 4. Materials and Methods

### 4.1. Isolation and Identification of K. pneumoniae Strains

#### 4.1.1. Isolation of *K. pneumoniae* Strains

Seventy samples were isolated from soil-dwelling invertebrates between September 2021 and March 2022. Digestive tracts were obtained from *Archispirostreptus syriacus* (millipede), *Porcellio laevis* (swift woodlouse), and *Armadillidium* sp. (isopods). The bacterial isolates were obtained using the dilution method, where gut contents were diluted and spread on nutrient agar media and incubated for 24 h at 37 °C. Morphologically identified *Klebsiella* isolates were also genetically identified using 16S rDNA sequencing.

#### 4.1.2. Application of 16S rDNA Gene Sequencing

Genomic DNA was isolated from all *K. pneumoniae* isolates using a DNA extraction kit (Gena Bioscience, Jena, Germany), according to the manufacturer’s instructions. One fragment of the DNA (approximately 1465 bp) was amplified from the 16S rDNA gene [30]. The pieces were punctuated using a QIAquick PCR purification kit (QIAGEN, Valencia, CA, USA) and sequenced using a DNA Analyzer 3146 Applied Bioscience (Applied Biosystems, Waltham, MA, USA). The sequencing texts were edited and compiled using the DNASTAR software (Laser gene 17.3, Madison, WI, USA). BLAST searches were performed using the National Center for Biotechnology Information server (http://www.ncbi.nlm.nih.gov/blast/Blast.cgi accessed on 7 March 2023).

#### 4.1.3. Antimicrobial Susceptibility Test

The antibiotic sensitivity of *K. pneumoniae* strains was studied using the disc diffusion method, according to Clinical and Laboratory Standards Institute (CLSI) guidelines [46]. This study was carried out using 12 commercially available antibiotics: sulfamethoxazole/trimethoprim (25 µg), ampicillin (10 µg), carbecillin (100 µg), amkacillin (30 µg), cefatrizine (10 µg), oxacillin (5 µg), penicillin (10 µg), ciprofloxacin (5 µg), gentamicin (10 µg), cefoxitin (30 µg), erythromycin (15 µg), and amoxicillin/clavulanic acid (30 µg).

#### 4.1.4. Detection of Virulence and Antibiotic Resistance Genes of *K. pneumoniae*

Twelve PCR reactions were performed to detect the presence of virulence genes (*acrAB*, *tolC*, *mdtk*, *Ompk35*, *fimH*, *rmpA*, *aea*, *k1*, and *k2*) in *K. pneumoniae* isolates [5], and antibiotic resistance genes, primer sequences, amplification conditions, and amplicon sizes were used as explained [20]. PCR was performed using the GoTaq^®^ Green Master Mix (Promega, Maddison, WI, USA). The expected sizes of the amplicons were ascertained by electrophoresis on 1.5% agarose gel with an appropriate molecular size marker (100 bp DNA ladder, MBI, Fermentas, LT, USA).

### 4.2. Leaf Extraction of R. stricta, HPLC Analysis, and Antibacterial Activity

#### 4.2.1. *R. stricta* Leaves Collection and Extraction Procedure

Fresh leaves of *R. stricta* were collected in September 2021 from their natural habitat at Taif-Makkah Road. The plant’s fresh leaves were air dried and ground into fine powder, then extracted using 100 mL of 95% ethanol and methanol separately at room temperature for 3 days. Each extract was centrifuged at 7000 rpm for 15 min and filtered 3 times with Whatman filter paper No. 1 to obtain a pure filtrate. The filtrate was passed through a Buchner funnel using a rotary vacuum evaporator (Dai-Han Inc., Seoul, Republic of Korea) at 30 °C, then the extracts (pellets) were dissolved in an aqueous solution of dimethylsulfoxide 1% (DMSO) [47]. The extracts were subjected to HPLC analysis to separate their components.

#### 4.2.2. HPLC (High Performance Liquid Chromatography) Analysis

Phenolic compounds were detected in the tested extracts as previously described [47], with fine modifications, using an Agilent 1260 infinity HPLC Series (Agilent, Santa Clara, CA, USA) equipped with a quaternary pump. Kinetex^®^ 5 µm EVO C18 100 mm × 4.6 mm (Phenomenex, Torrance, CA, USA) was used as the column and operated at 30 °C. The separation was carried out using a ternary linear elution gradient with (A) HPLC grade water with 0.2% and H_3_PO_4_ (*v*/*v*), (B) methanol, and (C) acetonitrile. Subsequently, 20 µL of the extract was injected. The *AVWD* detector (Agilent, Santa Clara, CA, USA) was set at 284 nm to detect phenols and flavonoids. 

#### 4.2.3. Antibacterial Activity of *R. stricta* Extracts

##### Disc Diffusion

The antibacterial properties of the *R. stricta* leaf extracts were assessed in triplicate using the agar disc diffusion method [23]. *K. pneumoniae* cells were allowed to grow for 24 h at 37 °C in a Mueller–Hinton liquid medium. The *K. pneumoniae* suspension was prepared in saline water, adjusted to 0.5 turbidity standards, and distributed in Mueller–Hinton agar (MHA, Oxoid, Basingstoke, UK). A sterile filter disc was impregnated with *R. stricta* leaf extract (10 μL/disc) placed on the agar surface. The MHA plates were kept for 2 h at 4 °C before their incubation at 37 °C for 24 h. The antimicrobial properties were categorized by measuring the zone of cell growth inhibition around the discs. The inhibitory activity was evaluated as previously described [26].

##### Determination of Minimal Inhibitory Concentrations (MICs) and Minimal Bactericidal Concentrations (MBCs)

MIC is the lowest concentration of the extract at which the growth of *K. pneumoniae* cells is inhibited. However, MBCs have the lowest concentrations of the extract that killed ≥ 99.9% of the initial *K. pneumoniae* cells. MIC and MBC were carried out 3 times using a 96-well microtiter plate (Nunc, Roskilde, Denmark) [29]. The *K. pneumoniae* suspension was prepared from an overnight culture diluted to 0.5 McFarland. Then, serial dilutions of both methanolic and ethanolic *R. stricta* leaf extracts were prepared in 5 mL of nutrient broth with concentrations extended from 0.012 to 50 mg/mL. Microtiter plates were prepared by placing 95 μL of nutrient broth and 5 μL of the *K. pneumoniae* inoculum in them, in addition to 100 μL of the respective dilutions of the extracts. The negative control contained 5 μL of bacterial inoculum and 195 μL of nutrient broth without the *R. stricta* extract. After incubation of the plates at 37 °C for 18–24 h, the MIC and MBC were determined [28]. MBC was determined by subculturing 20 μL of the clear wells of the MIC test on MHA. 

### 4.3. Biofilm Formation and Inhibition

#### 4.3.1. Biofilm Formation

The potential of *K. pneumoniae* strains to develop biofilms on U-bottomed, 96-well, microtiter polystyrene plates was tested using a crystal violet assay [28]. Briefly, *K. pneumoniae* cells were grown in a Trypticase Soy broth (TSB) media overnight at 37 °C. Then, 200 μL of the diluted culture (1:100) in TSB, supplemented with 2% (*w*/*v*) glucose, was transferred to a microtiter plate with wells containing sterile TSB as controls. After 24h of incubation at 37 °C, the cultures were removed, and the plates were washed 2 times with phosphate buffer saline and dried in an inverted position. Adherent cells were fixed with 95% ethanol and stained with 100 μL of 1% crystal violet (Merck, Lyon, France) for 5 min. The wells were then washed with 300 μL of sterile distilled water and left to dry in air. The biofilm produced was determined. 

#### 4.3.2. Biofilm Inhibition

*R. stricta* leaf methanolic and ethanolic extracts were tested for their ability to reduce the development of biofilms by *K. pneumoniae* isolates at MIC. In total, 100 µL of the extracts in TSB (2% glucose) were added to microtiter plate wells containing 100 μL of bacterial suspension (10^8^ CFU/mL) in each well. The negative control wells contained tryptic TSB and sterile water. Biofilm formation was determined using the crystal violet assay [28]. The percentage biofilm inhibition was calculated [47].

% Inhibition = 100 − ((OD570 sample)/(OD570 control) × 100)

This analysis was repeated three times.

### 4.4. Statistical Analysis

Three replicates were used for each of the treatments, and in each replicate, at least four plants were used, and the significance of the difference between the mean values was calculated. One-way analysis of variance (ANOVA) was used to perform the analysis of all data, and the significance of the difference among the treatments was determined according to the least significant difference (LSD) [47].

## 5. Conclusions

Soil invertebrates are important organisms harboring a lot of internal microflora in their digestive tract that need to be intensively studied. They already have useful microflora for the soil, but they may harbor pathogenic bacteria through their feeding habits, which may be harmful for humans. Therefore, we used leaf extracts of the wild plant *R. stricta* against the pathogen *K. pneumoniae*. Strong biofilm inhibitory activity and interesting antibacterial characteristics were shown by the extracts against *K. pneumoniae* isolates. *R. stricta* leaf extracts may be useful for treating or preventing *K. pneumonia* infections.

## Figures and Tables

**Table 1:** Source and locations of *Klebsiella pneumoniae* that were isolated from some invertebrates in Taif, Saudi Arabia.

Isolates	Species	Source	Locations
KTU-1	*Klebsiella pneumoniae*	millipedes	Shafa, Taif
KTU-2	*Klebsiella pneumoniae*	millipedes	Shafa, Taif
KTU-3	*Klebsiella pneumoniae*	millipedes	Shafa, Taif
KTU-4	*Klebsiella pneumoniae*	soft isopods	Hawia, Taif
KTU-5	*Klebsiella pneumoniae*	soft isopods	Hawia, Taif
KTU-6	*Klebsiella pneumoniae*	soft isopods	Hawia, Taif
KTU-7	*Klebsiella pneumoniae*	soft isopods	Hawia, Taif
KTU-8	*Klebsiella pneumoniae*	soft isopods	Hawia, Taif
KTU-9	*Klebsiella pneumoniae*	hard isopods	Hawia, Taif
KTU-10	*Klebsiella pneumoniae*	millipedes	Wady Ghazal, Taif
KTU-11	*Klebsiella pneumoniae*	millipedes	Wady Ghazal, Taif
KTU-12	*Klebsiella pneumoniae*	millipedes	Wady Ghazal, Taif
KTU-13	*Klebsiella pneumoniae*	millipedes	Shafa, Taif
KTU-14	*Klebsiella pneumoniae*	millipedes	Shafa, Taif
KTU-15	*Klebsiella pneumoniae*	millipedes	Shafa, Taif

**Table 2:** NCBI BLAST query for *Klebsiella pneumoniae* isolated from invertebrates in Taif, Saudi Arabia.

Isolates	Species	Query Coverage%	E Value	Ident%	Accession Number	Reference Accession No.
KTU-1	*Klebsiella pneumoniae*	100.00	0.00	99.00	ON077036	MN314310
KTU-2	*Klebsiella pneumoniae*	100.00	0.00	100.00	ON077037	MT349064
KTU-3	*Klebsiella pneumoniae*	99.00	0.00	99.00	ON077038	MN749610
KTU-4	*Klebsiella pneumoniae*	100.00	0.00	100.00	ON077039	MF076897
KTU-5	*Klebsiella pneumoniae*	99.00	0.00	99.00	ON077040	MF076897
KTU-6	*Klebsiella pneumoniae*	100.00	0.00	100.00	ON077041	MF076897
KTU-7	*Klebsiella pneumoniae*	99.00	0.00	100.00	ON077042	MF076897
KTU-8	*Klebsiella pneumoniae*	100.00	0.00	99.00	ON077043	MF076897
KTU-9	*Klebsiella pneumoniae*	100.00	0.00	99.00	ON077044	MF076897
KTU-10	*Klebsiella pneumoniae*	99.00	0.00	100.00	ON077045	MT349064
KTU-11	*Klebsiella pneumoniae*	98.00	0.00	99.00	ON077046	MF076897
KTU-12	*Klebsiella pneumoniae*	100.00	0.00	99.00	ON077047	MF076897
KTU-13	*Klebsiella pneumoniae*	100.00	0.00	99.00	ON077048	MF076897
KTU-14	*Klebsiella pneumoniae*	99.00	0.00	100.00	ON077049	MT349064
KTU-15	*Klebsiella pneumoniae*	100.00	0.00	100.00	ON077050	MN749610

**Table 3:** Antibiotic resistance profile of *Klebsiella pneumoniae* isolates.

Isolates	Antibiotic Profile
KTU-1	*Amp*,* Car*,* Caz*,* Oxa*,* Pen*,* Fox*,* Eth*,* Amc*
KTU-2	*Amp*,* Car*,* Caz*,* Oxa*,* Pen*,* Fox*,* Eth*,* Amc*
KTU-3	*Amp*,* Car*,* Caz*,* Oxa*,* Pen*,* Fox*,* Eth*,* Amc*
KTU-4	*Sxt*,* Amp*,* Car*,* Caz*,* Oxa*,* Pen*,* Fox*,* Amc*
KTU-5	*Amp*,* Car*,* Caz*,* Oxa*,* Pen*,* Fox*,* Amc*
KTU-6	*Car*,* Amk*,* Oxa*,* Pen*,* Fox*,* Eth*,* Amc*
KTU-7	*Car*,* Amk*,* Oxa*,* Pen*,* Fox*,* Eth*,* Amc*
KTU-8	*Car*,* Amk*,* Caz*,* Oxa*,* Pen*,* Fox*,* Amc*
KTU-9	*Amp*,* Car*,* Oxa*,* Pen*,* Fox*,* Eth*,* Amc*
KTU-10	*Car*,* Amk*,* Oxa*,* Fox*,* Eth*,* Amc*
KTU-11	*Car*,* Amk*,* Caz*,* Oxa*,* Pen*,* Fox*,* Eth*,* Amc*
KTU-12	*Car*,* Amk*,* Caz*,* Oxa*,* Pen*,* Fox*,* Eth*,* Amc*
KTU-13	*Sxt*,* Car*,* Amk*,* Caz*,* Oxa*,* Pen*,* Fox*,* Eth*,* Amc*
KTU-14	*Sxt*,* Car*,* Amk*,* Oxa*,* Pen*,* Fox*,* Eth*,* Amc*
KTU-15	*Sxt*,* Car*,* Amk*,* Oxa*,* Pen*,* Fox*,* Eth*,* Amc*

*Sxt* = sulfamethoxazole/Trimethoprim (25 µg), *Amp* = Ampicillin (10 µg), *Car* = Carbecillin (100 µg), *Amk* = Amkacillin (30 µg), *Caz* = Cefatrizine (10 µg), *Oxa* = Oxacillin (5 µg), *Pen* = Penicillin (10 µg), *Cip* = Ciprofloxacin (5 µg), *Gen* = Gentamicin (10 µg), *Fox* = Cefoxitin (30 µg), *Eth* = Erythromycin (15 µg), and *Amc* = Amoxicillin (30 µg).

**Table 4:** Virulence genes (*AcrAB*, *TolC*, *mdtk*, *Ompk35*, *FimH*, *RmpA*, *Aea*, *SHVM*, *TEM*, *K1*, and *K2*) in *Klebsiella pneumoniae* isolates.

Isolates	Virulence Genes
KTU-1	*AcrAB*,* TolC*,* mdtk*,* Ompk35*,* FimH*,* RmpA*,* Aea*,* SHV*,* TEM*
KTU-2	*AcrAB*,* TolC*,* mdtk*,* Ompk35*,* FimH*,* RmpA*,* Aea*,* SHV*,* TEM*
KTU-3	*AcrAB*,* TolC*,* mdtk*,* Ompk35*,* FimH*,* RmpA*,* Aea*,* SHV*,* TEM*
KTU-4	*AcrAB*,* TolC*,* mdtk*,* Ompk35*,* FimH*,* RmpA*,* Aea*,* SHV*,* TEM*
KTU-5	*AcrAB*,* TolC*,* mdtk*,* Ompk35*,* FimH*,* RmpA*,* SHV*,* TEM*
KTU-6	*AcrAB*,* TolC*,* mdtk*,* Ompk35*,* FimH*,* RmpA*,* Aea*,* SHV*,* TEM*
KTU-7	*AcrAB*,* TolC*,* mdtk*,* Ompk35*,* FimH*,* RmpA*,* Aea*,* K1*,* SHV*,* TEM*
KTU-8	*AcrAB*,* TolC*,* mdtk*,* Ompk35*,* FimH*,* RmpA*,* K1*,* SHV*,* TEM*,* CTX*
KTU-9	*AcrAB*,* mdtk*,* Ompk35*,* FimH*,* RmpA*,* K1*,* SHV*,* TEM*,* CTX*
KTU-10	*AcrAB*,* TolC*,* mdtk*,* Ompk35*,* FimH*,* RmpA*,* Aea*,* SHV*,* TEM*,* CTX*
KTU-11	*AcrAB*,* mdtk*,* Ompk35*,* FimH*,* RmpA*,* K1*,* SHV*,* TEM*
KTU-12	*AcrAB*,* TolC*,* mdtk*,* Ompk35*,* FimH*,* RmpA*,* Aea*,* SHV*,* TEM*
KTU-13	*AcrAB*,* TolC*,* mdtk*,* Ompk35*,* FimH*,* RmpA*,* Aea*,* SHV*,* TEM*
KTU-14	*AcrAB*,* TolC*,* mdtk*,* Ompk35*,* FimH*,* RmpA*,* Aea*,* SHV*,* TEM*
KTU-15	*AcrAB*,* TolC*,* mdtk*,* Ompk35*,* FimH*,* RmpA*,* Aea*,* SHV*,* TEM*

**Table 5:** Chemical compositions of *R. stricta* extracts (mg/kg).

Compounds	*R. stricta* Ethanolic Extract	*R. stricta* Methanolic Extract
Quinol	596.7	-
Resveratrol	823.35	1286.6
Chlorogenic acid	17.59	-
Vanillic acid	-	14.4
Caffeic acid	14.89	67.98
Syringic acid	58.4	109.5
p-Coumaric acid	662.9	10.43
Benzoic acid	1030.3	6334.8
Ferulic acid	424.6	1568.26
Ellagic acid	-	693,3
o-Coumaric acid	219.7	677.09
Cinnamic acid	139.6	445.06
Rosmarinic acid	200.59	485.3
Catechin	11.12	135.6
Rutin	793.8	1859.46
Quercitin	1256.7	2452.34
Neringein	316.15	8361
Myricetin	761.8	300.4
Kaempferol	7964.7	8249.13
Totals	15,292.89	33,050.65

**Table 6:** Antimicrobial activity of *Rhazya stricta* leaves extract against *Klebsiella* isolates.

*R. stricta* Extract	*Klebsiella* Isolates				
	(+ + + +) *n* (%)	(+ + +) *n* (%)	(+ +) *n* (%)	(+) *n* (%)	(−) *n* (%)
Ethanolic extract	6 (40.0)	5 (33.3)	3 (20.0)	1 (6.7)	-
Methanolic extract	5 (33.3)	4 (26.7)	4 (26.7)	2 (13.3)	-

Strong inhibitory action (+ + + +), Complete inhibitory action (+ + +), Partial inhibitory action (+ +), Slight inhibitory action (+), and No inhibitory action (−); *n*: number of isolates.

**Table 7:** Antibiofilm potentialities of ethanolic and methanolic *Rhazya stricta* leaf extracts against *Klebsiella* isolates.

Isolates	Biofilm Formation OD570 ± SD	Ethanolic Extract OD570 ± SD	Inhibition (%)	Methanolic Extract OD570 ± SD	Inhibition (%)
KTU-1	0.812 ± 0.081	0.150 ± 0.006 *	81.5	0.169 ± 0.073 *	79.2
KTU-2	0.448 ± 0.102	0.034 ± 0.021 *	92.4	0.195 ± 0.103 *	56.4
KTU-3	0.588 ± 0.319	0.031 ± 0.009 *	94.7	0.084 ± 0.011 *	85.7
KTU-4	0.808 ± 0.109	0.036 ± 0.019 *	95.5	0.192 ± 0.146 *	76.2
KTU-5	0.744 ± 0.286	0.070 ± 0.031 *	90.5	0.149 ± 0.057 *	79.7
KTU-6	0.799 ± 0.818	0.089 ± 0.024 *	88.9	0.123 ± 0.002 *	84.6
KTU-7	0.625 ± 0.350	0.027 ± 0.014 *	95.6	0.132 ± 0.106 *	78.8
KTU-8	1.015 ± 0.158	0.028 ± 0.024 ***	94.3	0.211 ± 0.089 **	79.2
KTU-9	0.987 ± 0.025	0.072 ± 0.046 *	91.1	0.265 ± 0.063 **	67.2
KTU-10	1.053 ± 0.041	0.120 ± 0.066 **	97.2	0.237 ± 0.230 **	85.8
KTU-11	0.971 ± 0.226	0.042 ± 0.079 **	95.7	0.204 ± 0.253 **	78.9
KTU-12	0.802 ± 0.444	0.038 ± 0.016 *	95.2	0.194 ± 0.260 *	75.8
KTU-13	1.060 ± 0.006	0.014 ± 0.132 ***	98.7	0.206 ± 0.097 **	80.4
KTU-14	0.422 ± 0.115	0.030 ± 0.001 *	92.8	0.274 ± 0.385 *	35.1
KTU-15	0.631 ± 0.014	0.037 ± 0.001 *	94.1	0.190 ± 0.111 *	69.8

* Isolates changed from low-grade positive to biofilm-negative. ** Isolates changed from highly positive to low-grade positive. *** Isolates changed from highly positive to biofilm-negative.

## Data Availability

Not applicable.
